# CoAP-Based Mobility Management for the Internet of Things

**DOI:** 10.3390/s150716060

**Published:** 2015-07-03

**Authors:** Seung-Man Chun, Hyun-Su Kim, Jong-Tae Park

**Affiliations:** School of Electronics Engineering, College of IT Engineering, Kyungpook National University, Daegu 702-701, Korea; E-Mails: smchun@ee.knu.ac.kr (S.-M.C.); hs_kim@ee.knu.ac.kr (H.-S.K.)

**Keywords:** IoT mobility management, web-based mobility management, WoT mobility management

## Abstract

Most of the current mobility management protocols such as Mobile IP and its variants standardized by the IETF may not be suitable to support mobility management for Web-based applications in an Internet of Things (IoT) environment. This is because the sensor nodes have limited power capacity, usually operating in sleep/wakeup mode in a constrained wireless network. In addition, sometimes the sensor nodes may act as the server using the CoAP protocol in an IoT environment. This makes it difficult for Web clients to properly retrieve the sensing data from the mobile sensor nodes in an IoT environment. In this article, we propose a mobility management protocol, named CoMP, which can effectively retrieve the sensing data of sensor nodes while they are moving. The salient feature of CoMP is that it makes use of the IETF CoAP protocol for mobility management, instead of using Mobile IP. Thus CoMP can eliminates the additional signaling overhead of Mobile IP, provides reliable mobility management, and prevents the packet loss. CoMP employs a separate location management server to keep track of the location of the mobile sensor nodes. In order to prevent the loss of important sensing data during movement, a holding mode of operation has been introduced. All the signaling procedures including discovery, registration, binding and holding have been designed by extending the IETF CoAP protocol. The numerical analysis and simulation have been done for performance evaluation in terms of the handover latency and packet loss. The results show that the proposed CoMP is superior to previous mobility management protocols, *i.e.*, Mobile IPv4/v6 (MIPv4/v6), Hierarchical Mobile IPv4/v6 (HMIPv4/v6), in terms of the handover latency and packet loss.

## 1. Introduction

The Internet of Things (IoT) enables real world objects to be integrated into a virtual world, where sensors, actuators, and other devices interact not only with human users, but also with each other and software agents on the Internet. One approach for making IoT data available to users is the use of Web service technologies, which can directly integrate IoT data and Web functionalities through the Internet. This integration of Web services with IoT has been defined as the Web of Things (WoT) [1]. Furthermore, sensors in IoT environment have been miniaturized, integrating various communication functions, such as Bluetooth, ZigBee, Low-power WiFi, and GPS. 

The Internet Engineering Task Force (IETF) has undertaken much standardization work related to WoT. For example, the IETF Constrained RESTful Environments (CoRE) Working Group (WG) has been creating standardizations for introducing the Web service paradigm into networks of smart objects. The CoRE WG has defined a REST-based Web transfer protocol, the Constrained Application Protocol (CoAP) [2]. CoAP can make it easy to integrate physical devices with contents on the Web, while satisfying requirements, such as multicast support, low signaling overhead, and simplicity for constrained network environments. The devices in a constrained network environment generally tend to be embedded, and to require considerably less CPU processing, memory, and power supply capabilities than Internet devices. More specifically, the constrained node often have 8-bit microcontrollers with small amounts of ROM and RAM, while constrained networks such as IPv6 over Low-Power Wireless Personal Area Networks (6LoWPANs) often have high packet error rates and a typical throughput of 10 s of kbit/s. Therefore, the requirements of multicast support, low signaling overhead, and simplicity are extremely important in a WoT environment. In addition, the constrained networks in a WoT environment usually have a limitation in packet size, may exhibit a high degree of packet loss, and may have a substantial number of devices in sleep mode operation [2]. In a duty-cycled network, sensor nodes perform four distinct operational states: transmit, receive, idle, and sleep. In sleep states, the sensor is shut down and a low-power timer is on to wake up the sensor at a later time [3]. Therefore, it can consume only a tiny fraction of the energy consumed in the active mode. 

The interaction model of CoAP is similar to the client/server model of HTTP. However, unlike HTTP, the end-points of the CoAP may act as both clients and servers [2,4]. The architecture of CoAP is divided into two layers: message and request/response. CoAP supports reliable message transmissions by using stop-and-wait retransmissions with an exponential back-off mechanism to correct the order of packets and check duplicates [1]. CoAP can benefit various IoT applications, such as ubiquitous healthcare services, V2I/V2V automatic vehicle networks, home networks, automotive networks, automatic systems, industrial networks, interactive toys, and remote meters.

One limitation of CoAP is that it does not directly address the mobility requirements for mobile constrained nodes in WoT environments. CoAP has been designed for Machine to Machine (M2M) applications, such as smart energy and building automation [5]. In previous WoT applications, WoT researchers assumed that most sensor nodes do not have movement. Therefore, the sensor mobility has not been considered in a WoT network environment. A sensor node, however, can have mobility. For example, in a vehicle monitoring system, the vehicle can move into different IP domains. In the ubiquitous network, the characteristics of the wireless network or sensor movement can change the wireless connection between the sensor and wireless access router. In WoT environment, the sensor provides the sensing resource and acts as Web server. In this paper, CoAP node indicates its sensor, which can provides the sensing resource, responses the request of another client, and is equipped CoAP protocol in the constrained network environment. As a CoAP sensor moves around different wireless networks, its IP connectivity may be disrupted, which may result in the loss of important sensing data, or delay of time-critical data. In CoAP, the IP address of CoAP node, is registered with the Domain Naming Server (DNS). The DNS configuration involves operation by humans as much as possible. If the CoAP server node moves between different IP domains, the client may not find the server location, *i.e.*, IP address, if the human may not configure the changed IP address on DNS in time. 

To prevent the previously described mobility problem of a Web server node, an existing mobility management protocol may be used. The IETF has developed various standard mobility management protocols. The mobility management for network layer, Mobile IPv4/v6 (MIPv4/v6) [6] and its variants, including Fast Mobile IPv4/v6 (FMIPv4/v6) [7], Hierarchical Mobile IPv4/v6 (HMIPv4/v6) [8], and Proxy Mobile IPv4/v6 (PMIPv4/v6) [9] were developed. The transport layer uses TCP migrate and the mobile Stream Control Transmission Protocol [10]. For the application layer, SIP-based approaches [11] have been proposed to manage mobility in next-generation wireless networks. 

Unfortunately, most standard mobility management protocols add high signaling overhead from tunneling and binding operations, and are quite complex, incurring high processing and energy consumption. Additionally, most standard mobility management protocols require the modification of the network infrastructure such as Internet access router and mobile nodes. Furthermore, these standard mobility management protocols do not address the characteristics of a constrained IP network, such as limitations in packet size, high packet loss ratio, and sleep mode operation. Therefore, the protocols mentioned previously may not be suitable for mobility management in a WoT environment with constrained device and network characteristics; for example, with low processing and energy constraints, or in sleep mode operation. 

With regard to the objective functionality of mobility management, the objective of a WoT environment differs from that of existing IETF mobility management protocols. More specifically, in conventional IETF mobility management protocols, the objective of mobility management is to enable a mobile node to initiate a session and be provided with an application service in a seamless manner during an IP handover. In a WoT environment, however, the objective is to enable a mobile sensing node to timely send measured data to a remote client whenever the client requests it. Therefore, a WoT environment needs a novel mobility management protocol that can satisfy the previously described objective, considering constraints on processing capability, energy consumption, and other characteristics, such as sleeping mode operation. 

Jara *et al.* presented a lightweight Mobile IPv6 with IPSec, which is aware of the requirements of the IoT and analyzes the efficiency and security adapted to IoT-devices capabilities [12,13]. The authors proposed the lightweight Mobile IPv6, which does not execute the route optimization and return routability of the original MobileIPv6, to be integrated into constrained devices with a low capacity in terms of memory and communication capabilities. Additionally, the authors investigated the requirements for supporting the mobility management in IoT environment [12]: global identifiers, IPv6-based protocol, communication costs, packet encapsulation, and movement detection. In the lightweight Mobile IPv6, the home agent and foreign agent play a role as middle agent in order to deliver the ingoing packet, *i.e.*, control packet and real packet to the mobile node or corresponding node. As a result, the load of middle agent can be dramatically increased when the number of mobile nodes increases and the triangular routing problem can be incurred. Hence, the process of control packet and real packet may be separated. This lightweight MobileIPv6 does not consider the sleep mode operation of IoT devices and requires the modification of infrastructure such as home agent and foreign agent because it is based on Mobile IPv6.

Sungmin *et al.* proposed the Sensor Networks for an All-IP World (SNAIL) based on MARIO [14]. In this research, the sensor is composed of PAN coordinator, static node, partner node, mobile node, and gateway. SNIL uses the ancestral concept to perform the handover. More specifically, the mobile node retrieves the domain information of next static node, *i.e.*, node ID and IP address, through the partner node before the mobile node performs the handover. After that, in the next domain, the mobile node performs the binding update with next domain information. As a result, the handover delay can be reduced. However, as the mobile node does not move into pre-defined location of next static node, the handover delay and packet loss can be large. Also, a PAN coordinator may always manage and update the information of near sensor. It can occur a large signaling overhead in the network domain.

Jara *et al.* presented a protocol to carry out inter-WSN mobility inside of the architecture that has been defined at a hospital [15]. It can decrease the number of interchanged messages of mobile nodes when the mobile nodes move within pre-defined regions. However, it is not suitable for IoT global mobility protocol because this mobility protocol cannot support the global mobility and the modification of network infrastructure is required. Kai *et al.* presented the Care-of Address Pool for Hierarchical MIPv6 (CoAP-HMIPv6) to reduce the handover latency by reducing influence caused by the DAD procedure [16]. However authors have not considered the mobile network with the constrained resource. Gligoric *et al*. have proposed the Open Mobile Alliance device management protocol for reliable Device Management (OMA-DM) and have analyzed and compared the efficient XML interchange (EXI), CoRE Link format, and protobuf for efficient message format [17]. The authors proposed EXI is efficient as the payload format in use of CoAP. 

Berguiga *et al.* presented a mobility management scheme for 6LoWPAN sensor nodes [18]. The authors proposed the fast handover proxy mobile IPv6 for sensor network (FPMIPv6 S) protocol, an improved version of the Proxy Mobile IPv6 (PMIPv6) protocol, to reduce the number of messages exchanged and the handover latency. However, they did not consider the complexity of FMIPv6, with respect to CPU processing overhead and energy consumption.

Ganz *et al.* presented a resource mobility scheme for service continuity in an IoT environment [19]. They proposed a resource mobility scheme using two operating modes, caching and tunneling, to enable applications to access the sensory data when a resource becomes temporarily unavailable. The sensor gateway caches the measured data, and transmits the data in response to a service provider’s request instead of the sensor. The tunneling method reduces the amount of packet loss during the handover of a sensor by creating a tunnel between the sensor gateways. However, as both sensor gateway and sensor itself can move between different wireless networks, the connectivity might be disrupted during their movement. 

In summary, most current mobility management protocols may not be suitable for supporting the mobility of CoAP sensor nodes in WoT environments because the sensor nodes in such an environment generally have constrained CPU processing power and memory capacities and they must have low energy consumption. They have other characteristics such as sleep mode operation and a constrained network of wireless sensor networks. Current mobility management standards of the IETF have not addressed these constraints on the design of mobility management architecture and protocols. 

In this article, we propose the CoAP-based Mobility Management Protocol (CoMP), which can provide mobility management for mobile CoAP sensor nodes. Because CoMP uses a separate location management function, which is based on CoAP, low signaling overhead can be obtained due to simplicity of the mobility management architecture. The tunneling scheme is not used for architectural simplicity. CoMP enables the IP addresses of mobile CoAP sensor nodes to be kept track of, allowing monitored sensing data to be reliably delivered to Web clients using both HTTP and CoAP. To the best of our knowledge, there have been no previous research attempts at providing direct IP mobility functionality to mobile CoAP nodes. Compared with other related works, the originality of our approach may be summarized as follows:

• Instead of designing new signaling protocols for mobility management, CoMP employs the IETF standard CoAP protocol for mobility management in an application layer, without changing the lower layer. This achieves the simple seamless connectivity of wireless constrained sensor node without the modification of the existing network infrastructure.
• CoAP messages and methods are extended to implement the mobility management functions of a mobile CoAP node, which imparts not only simplicity in the mobility management architecture, but also has significantly low signaling overhead, compared to other protocols, such as MIPv4/v6 and its variants.
• In the existing IETF MIPv6 mobility management protocol, a bi-directional tunnel scheme has been used for transparent handover operation. Instead of a bi directional tunnel, CoMP uses two modes of operation, holding and binding, for fast and reliable data transmission.

The contributions of our research are as follows:

• The detailed architecture and functions of CoMP have been designed for mobility management. A separate location management function to support CoAP service mobility has been designed.
• The sleep mode operation of sensor node in CoMP is considered to provide reliable service.
• Detailed signaling procedure and an address management method were designed for supporting seamless connectivity and reliable transmission.
• To enhance interoperability, we extended CoAP; more specifically, CoAP messages and methods were extended to exchange messages for managing IP addresses between CoAP nodes.

The remainder of this paper is organized as follows. In Section 2, we describe the overview and limitations of the CoAP standard. We also describe the comparison of CoMP with the existing standard mobility management protocols such as Mobile IP and SIP-MM. In Section 3, we describe the architecture and message formats of the proposed CoMP. In Section 4, we present a mathematical analysis of the proposed CoMP handover mechanism for a performance evaluation. In Section 5, we describe the performance results of the proposed scheme. Finally, in Section 6, we provide some concluding remarks regarding this research.

## 2. Background 

In this section, the overview of CoAP and its limitation are introduced. The comparison of CoMP with the existing mobility management protocols such as Mobile IP and SIP-MM are also given. 

### 2.1. Overview of CoAP and Its Limitation

The IETF CoRE WG [2] has designed CoAP for resource oriented applications intended to run on constrained IP networks. These networks and the nodes within them have severe limits on throughput, available power, and in particular, the amount of complexity that can be supported with a limited code size and limited RAM size per node [2]. For example, sensor nodes often have 8-bit microcontrollers with small amounts of ROM and RAM; while constrained networks, such as 6LoWPAN often have high packet error rates (5%–10% is common) and a typical throughput of 10 kbit/s. 

CoAP, based on an asynchronous request/response interaction model between application endpoints, supports built-in discovery of services and resources, and includes key Web concepts, such as Uniform Resource Identifiers (URIs) and Internet media types [2]. The server and client correspond to sensing nodes and Web clients, respectively. The resource information of the server such as the URI and IP address, is published at the Web Application Description Language (WADL) server [20]. The client can retrieve and access the measured data of the sensor node by referring to the resource information on the WADL server.

**Figure 1:**
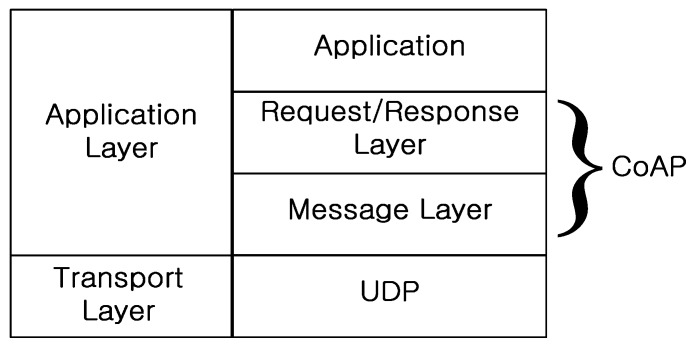
CoAP architecture.

Figure 1 shows CoAP architecture, which consists of two layers: message and request/response. The function of the CoAP message layer is to control message exchanges over UDP between two endpoints. There are four message types: *confirmable* (CON), *non-confirmable* (NON), *acknowledgement* (ACK), and *reset* (RST). 

At the request/response layer, CoAP request and response semantics are carried in a message, and include either a method code or a response code. The message also carries optional information, such as the URI and type of payload content. CoAP can match the requests and responses using a message ID, and a token option differentiates concurrent requests. The characteristics of CoAP are summarized below.

• **Compact header**: CoAP includes a compact binary header with extensible options. The protocol has a base header size of only 4 bytes, and a total header of 10–20 bytes for a typical request.
• **Methods and URIs**: For a client to access server resources, CoAP supports the GET, PUT, POST, and DELETE request methods. These methods are answered using a subset of HTTP-compatible response codes. CoAP supports URIs, which are a key feature of the Web architecture.
• **Simple caching**: Caching for resource representations is supported to optimize performance in constrained network environment.
• **Asynchronous message exchanges:** The messages that are exchanged between the client and the server are operated asynchronously.

These CoAP functional characteristics are implemented under the assumption that the IP address of the constrained sensing node is fixed. As the IP address of the server node may change because of node movement, the client may not find the location of the corresponding server node. As a result, the client may not retrieve the time-critical data from the server. To ensure timely data availability in a WoT environment, a simple and efficient mobility management function should be provided to handle server mobility.

### 2.2. Comparison with Standard Mobility Management Protocols 

To support the mobility management protocol, several mobility protocols have been proposed for wireless Internet [5,6,7,8,9,10,11,12,13,14,15,16,17,18,19]. These protocols can be broadly classified based on the layer of their operation, such as those operating in the network layer, transport layer and application layer. The dependency of these mobility protocols on the access networks reduces progressively as we move up on the protocol stack. Among them, Internet Engineering Task Force (IETF) has standardized Mobile IP (MIP) and Session Initiation Protocol (SIP) as the mobility solution for the network layer and application layer, respectively.

Table 1 shows the comparison of CoMP with the Mobile IP and SIP MM. Mobile IP allows the mobile node to acquire and register a new IP address in each visited network. Mobile IP [6] is the main protocol for mobility management at the IP layer, which allows a mobile node to remain reachable despite of its movement within the IP environment. The Mobile IP uses the tunnel mechanism to prevent the packet loss during the handover between the access router and the mobile node or between access routers. Mobile IP, however, requires significant changes in the underlying networking infrastructure. Furthermore, the mobile node requires the routing function and tunneling function in order to support the tunneling scheme. In IoT network environment, the routing function and the tunneling function at the mobile node requires the high processing capability, high power consumption, high memory, etc. Mobile IP may not be suitable for IP mobility management at IoT/WoT networks.

SIP is an application level signaling protocol that controls communication sessions for multimedia flows in the Internet, such as voice or video calls. Through SIP’s name mapping and redirection services, it can be used for personal mobility. Application layer protocols however, are transparent to the lower layer characteristics and they maintain end-to-end semantics of a connection. The application layer protocols are also expected to be the right candidate for handling mobility in a heterogeneous environment. SIP is capable of supporting not only terminal mobility but also session mobility, personal mobility and service mobility. In addition, SIP can support IP mobility without the tunneling scheme. Therefore, SIP has been considered as an attractive candidate at the application layer mobility management protocol for heterogeneous 5G wireless networks [21]. 

**Table 1:** Comparison of CoMP with Mobile IP and SIP-MM.

Classification	Mobile IP	SIP-MM	CoMP
Modification of layer	Network	Application	Application
Signaling overhead	Large	Large	Small
Tunnel used	Bi-directional tunneling	No use tunnel	No use tunnel
Application type	Text, Multimedia	Multimedia, VoIP	Resource-based application(Sensing information)
Power consumption	Very high	High	Very low
Resource Provider	Remote Server	Remote server	Mobile sensor
Session initiation agent	Mobile node	User Agent (Mobile node)	Web client
Mobility management agent	Home agent	Remote server	Remote server
Session initiation agent	Mobile node	Mobile node	Web client
Retransmission mechanism of handover management message support	Not supported	Not supported	Supported (Stop-and-wait retransmissions with an exponential back-off mechanism)

However, SIP may not be suitable for IoT network environments with network constraints such as the limited packet size, low bandwidth and resource constraints such as low power, low CPU processing capacity, and small memory. In particular, SIP requires an additional application header to carry its signaling messages, and hence is limited by the performance of TCP or UDP over constrained wireless links. More specifically, in SIP, the maximum transmission unit (MTU) of a packet requires 1500 bytes. However, the MTU of IEEE 802.15.4 standard is limited to 127 bytes. Hence, SIP may not effectively provide the mobility management at IoT network environment.

In Mobile IP and SIP, the session initiation agent is the mobile node. On the contrary, in WoT network environment, the Web client performs the session initiation. In addition, the sensor node in IoT environment usually operates either in sleep or wakeup mode. Hence, the Web client may not get the required data from the senor node in sleep mode operation.

Therefore, the protocols mentioned previously may not be suitable for mobility management in a IoT environment with constrained device and network characteristics; for example, with low signaling overhead, low processing and energy constraints, or in sleep mode operation. Therefore, the novel mobility protocol is required to solve the previous problems.

To solve the mentioned previous problems, we propose the CoAP-based mobility management protocol, called CoMP, for IoT network environment. The salient feature of CoMP is that it can provide the fast and reliable IP handover of the sensor node without changing the lower layer, with low signaling overhead, and with no packet loss, while taking into account the sleep/wakeup mode of operation.

## 3. Mobility Management Architecture Using CoAP

In this section, we describe the mobility management architecture for a mobile CoAP node that is based on CoMP. We also describe the detailed mobility management procedure and message format of CoMP.

### 3.1. Mobility Management Architecture of CoMP 

**Figure 2:**
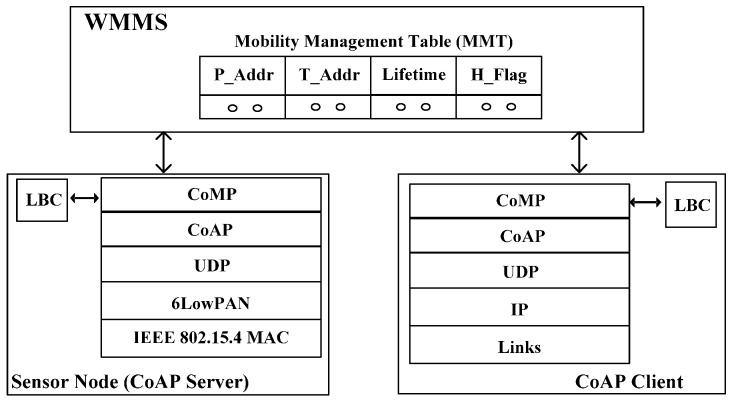
Mobility management architecture of CoMP.

Figure 2 shows the mobility management architecture using CoMP. The components of the architecture consist of a CoAP server and CoAP Client nodes and WoT Mobility Management System (WMMS) with a Mobility Management Table (MMT).

The CoAP client node may request the retrieval of data for the CoAP server node. The WMMS maintains the location address information in MMT, which is necessary to perform mobility management, keeping track of the location of a moving CoAP node. A CoAP node is usually a tiny sensor node that monitors the measured data, and transmits the measured data to the requesting node *i.e.*, CoAP client and Web client. 

In WMMS, P_Addr in MMT is the permanent IP address of the CoAP node that is registered at DNS. T_Addr in MMT is the temporary IP address of the CoAP node, which can be changed as the access point of the CoAP node is changed. H_Flag indicates the handover status of the node. If H_Flag is “1”, it indicates that the corresponding CoAP node is in handover status; therefore, it may not receive the packet from other nodes. H_Flag is “0”, it indicates that the corresponding node is not in handover status. A Lifetime is the time to which the binding of P_Addr with T_Addr is effective. The sleep period is sleep time in sleep mode operation of CoAP node. In this time, WMMS does not send a request message to CoAP node until the sleep period expires.

**Figure 3:**
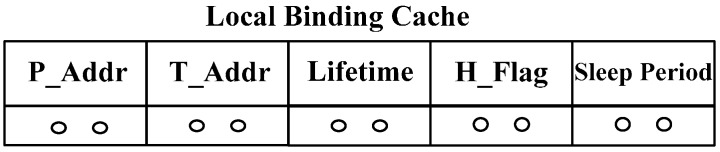
LBC schema.

As shown in Figure 2, the network architecture of a CoAP node is comprised of CoAP and CoMP at the application layer, UDP at the transport layer, a 6LoWPAN at the network layer, and IEEE 802.15.4 at MAC layer. CoAP is composed of the message layer and request/response layer. The CoMP makes use of GET, POST, PUT, and DELETE methods at the CoAP Request/Response layer in order to provide mobility management functionality. The CoAP node contains a local binding cache (LBC), which includes P_Addr, T_Addr, Lifetime, and H_Flag. In order to provide the mobility management function, the CoMP refers to the LBC table, whose schema is shown in Figure 3. The meanings of those fields on LBC are the same as those in MMT. The Lifetime value of “0” at LBC indicates that an LBC entry for the CoAP node must be deleted and retrieved from the entry of the CoAP node in the MMT of the WMMS. A key feature of CoMP is the use of hold mode, *i.e.*, H_Flag, to prevent packet losses while a CoAP node is moving among different wireless networks.

### 3.2. Mobility Management Procedure of CoMP 

**Figure 4:**
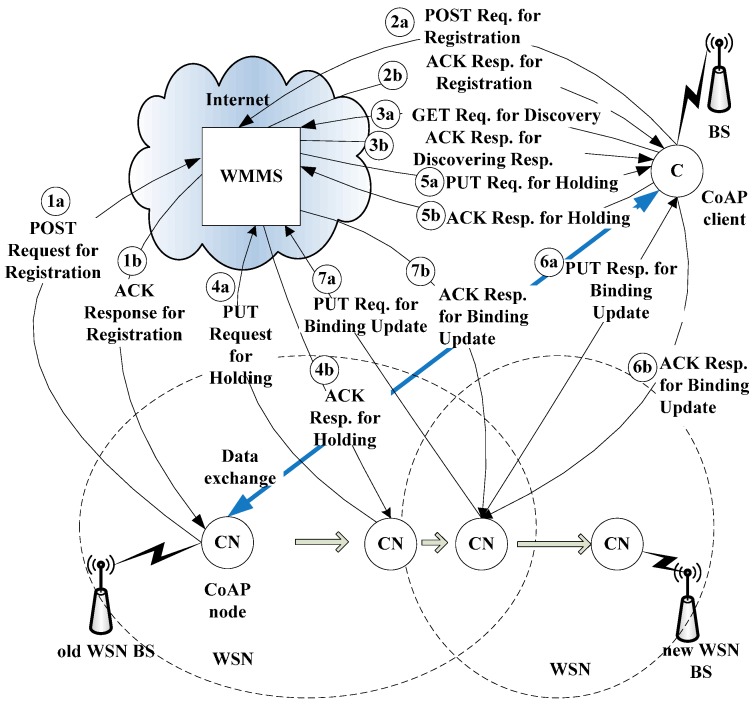
Mobility management procedure of CoMP.

Figure 4 shows the detailed mobility management procedure for IP mobility management. The CoMP consists of four procedures, *i.e.*, registration, discovery, binding, and notification, to provide mobility management for a moving CoAP node. The operation of CoMP is described in detail below. First, in Figure 4, both the CoAP client and CoAP node send the POST request message for registration to the MMS in order to register their own P_Addr and Lifetime in the MMT of the MMS. As the CoAP client attempts to communicate with the CoAP node, the CoAP client sends a GET request message to the MMS for discovery. This message includes the CoAP client’s destination IP address. In response, the CoAP client receives the current T_Addr for the CoAP node and its Lifetime in the ACK response message for discovery. Then, the CoAP client stores the T_Addr and Lifetime for the CoAP node in the LBC. Subsequently, the CoAP client can exchange data with the CoAP node directly until the Lifetime of T_Addr expires. 

Next, let us consider the case in which the CoAP node moves from the old base station (BS) such as router, access router to the new BS of the new WSN. As the CoAP node moves away from the old WSN BS and enters the network domain of the new BS, it requires the IP handover operation (as illustrated in Figure 4). In order to perform the handover operation, the CoAP node first detects the radio signal strength (RSS) from the old ER at the link layer. When the RSS from the old BS drops below a certain threshold value, the CoAP node prepares the handover operation. In order to prevent packet loss during the handover operation, the CoAP node notifies the CoAP client of its status—*i.e.*, handover mode—by sending a PUT request message to withhold access requests from the WMMS. The WMMS then updates the H_Flag of the CoAP node in the MMT to “1.” It also forwards the PUT request message so that requests from the CoAP Client are withheld. In response, the CoAP client likewise updates the H_Flag in its LBC to “1.” Because the H_Flag of a CoAP node indicates that the node is performing a handover operation—and consequently cannot be accessed. 

During a handover, the CoAP node resides in the overlapped region of two network domains: the old BS and the new BS. The CoAP node detects the movement of a CoAP node through the Router Advertisement (RA) and Router Solicitation (RS) messages. As soon as it detects the new BS network domain, the CoAP node attempts to secure a new temporary IP address—*i.e.*, T_Addr from the new ER—by using Neighbor Solicitation and Neighbor Advertisement.

After receiving a new T_Addr, the CoAP node notifies both the MMS and the Web client of its new T_Addr by using a PUT request message for a binding update. It also changes the H_Flag in its LBC and the MMT of the WMMS. The PUT request message for the binding update includes the P_Addr and T_Addr of the CoAP node, and the H_Flag status. After receiving the binding update message from the CoAP node, the CoAP client changes the H_Flag for the CoAP node to “0.” Finally, the CoAP client can retrieve the sensing data from the CoAP node. As a result, the CoAP client and the CoAP node can exchange data without packet loss during the handover.

As previously described in Section 1, a CoAP node may be in sleep mode operation. In sleep mode, a CoAP node may not send or receive the data. The CoAP node can wake up when it receives the beacon message from the WSN BS. Without being notified of the sleep mode of CoAP node, a CoAP client which tries to get a CoAP node in sleep mode, may retry to connect to the CoAP node in sleep mode, continuously. This may result to a large traffic overhead in WoT environment. Furthermore, if the CoAP node in active operation abruptly falls into the sleep mode due to the power shortage, the ongoing connection may be disrupted, and large packet loss may occur. 

In order to consider the sleep mode operation of CoAP node, we use the PUT request/response messages for holding using H_Flag in CoMP. More specifically, the CoAP node exchanges the PUT request/response messages to perform the hold mode operation with WMMS before the CoAP client goes to sleep mode operation. The PUT request message for holding includes the H_Flag and sleep period to reflect the status of CoAP node. When the WMMS receives the PUT request message for holding, WMMS holds on the request message until expires the sleep period of CoAP node. In a result, the PUT request/response messages for holding can prevent this unnecessary network traffic overhead and packet loss due to the sleeping CoAP server.

### 3.3. Message Format of CoMP 

**Figure 5:**
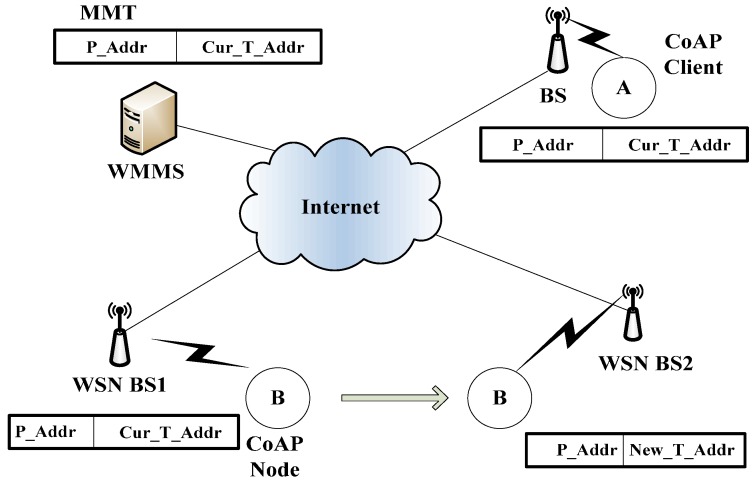
IP address change during CoMP handover.

**Figure 6:**
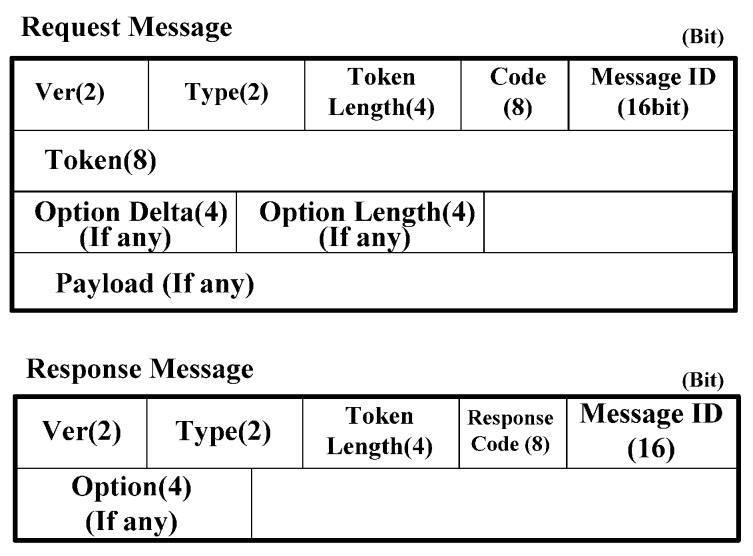
Request/Response message format.

In this subsection, we present the message format using CoAP. Figure 5 shows the IP address information during CoMP handover. We assume that the CoAP node moves from WSN BS1 to WSN BS2. In this situation, Cur_T_Addr and Cur_Lifetime of CoAP node are changed to New_T_Addr as a temporary IP address, *i.e.*, T_Addr, and New_Lifetime as Lifetime, respectively. However, P_Addr as the permanent IP address, *i.e.*, P_Addr, does not change. P_Addr, T_Addr, and Lifetime of CoAP nodes are cached on the WMMT of the WMMS. W_Addr indicates the IP address of the WMMS. Figure 6 shows the request message and response message format in the CoAP standard. The detailed information refers to the CoAP standard document [2]. The message format is based on the RESTful format.

In Figure 6, Ver indicates version and Type indicates the message type, *i.e.*, Confirmable (0), Non-confirmable (1), Acknowledgement (2), and Reset (3). The code indicates the message type of request or response, *i.e.*, GET (1), POST (2), PUT (3), and DELETE (4). The message ID indicates the identifier of the message, which is created by the sender of the CoAP node. The token is intended for use as a client-local identifier for differentiating between concurrent requests. A CoAP node should generate tokens in a way that tokens currently in use for a given source/destination pair are unique. In a response message, the response code indicates that the status code that the client requested was successfully received, understood, and accepted.

**Figure 7:**
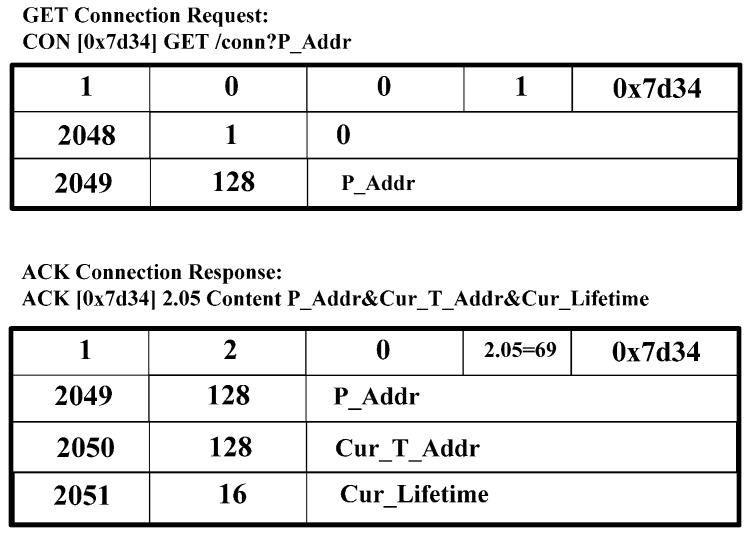
GET request, ACK response message for discovery of IP address.

**Figure 8:**
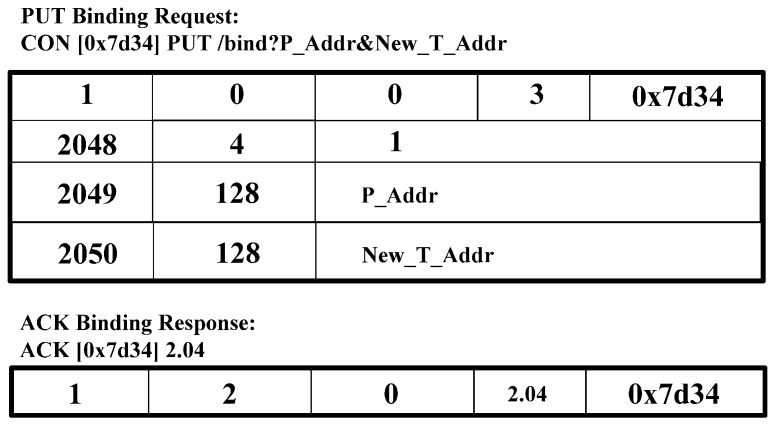
PUT binding request message and ACK binding response message for binding update.

**Figure 9:**
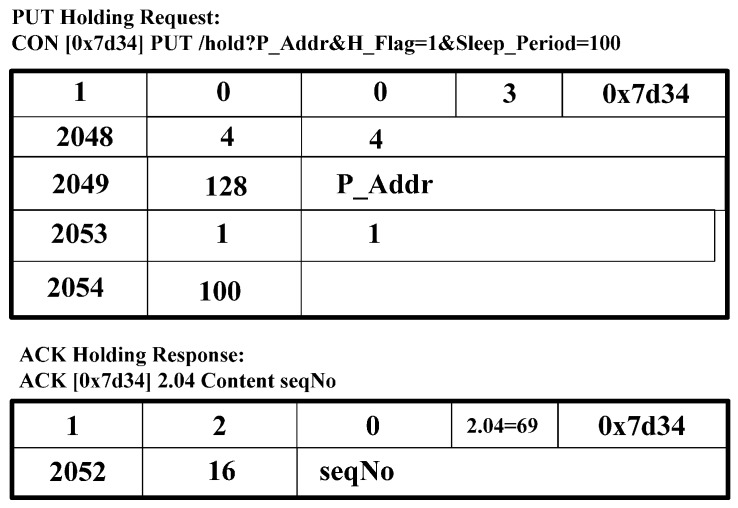
PUT request message and ACK response message for holding.

**Figure 10:**
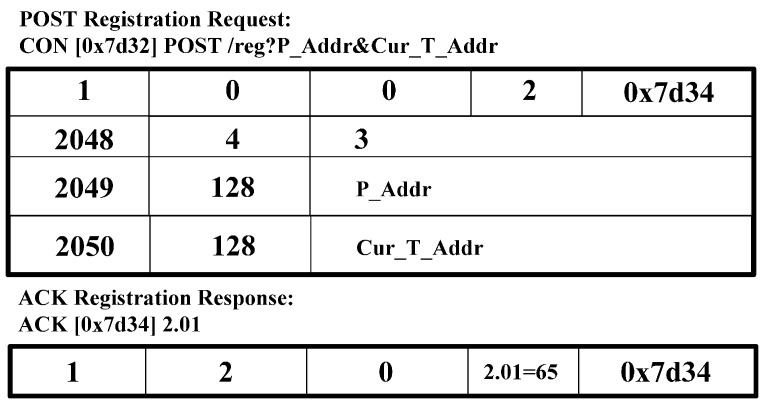
POST request and ACK response message for registration.

**Figure 11:**
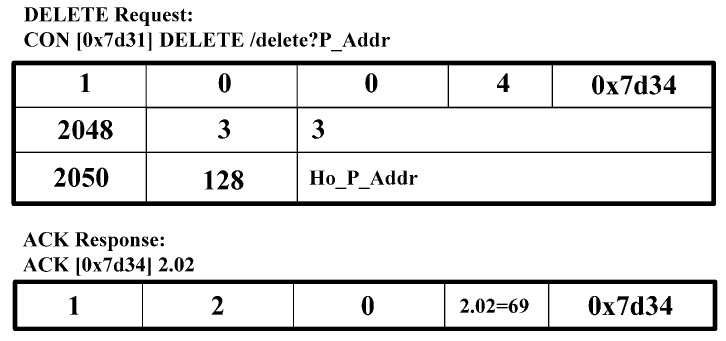
DELET0E request and ACK response message.

Figure 6 shows the CoAP message format [2]. We use the CoAP message format and extend the option delta to perform the CoMP signaling procedure. In the CoAP message format in Figure 6, we define the option delta and option length to specify the resource constraints. We extend the option delta value to support the CoMP. Table 2 shows the extended option delta and its description of the CoMP message. In the option delta, the 2048–2054 are newly defined. The range of 2048 to 64,999 of option delta gives the designated expert in CoAP standard [2]. The message type field includes CoAP methods, *i.e.*, GET, PUT, POST, and DELETE. Figure 7, Figure 8, Figure 9, Figure 10 and Figure 11 show the CoMP message format.

**Table 2:** Extended Option Delta and Descriptions for CoMP.

Option No.	Name	Format	Length(bits)	Comment
2048	Message type	Unit	1	This is used to specify the request-message type: “0” indicates a GET request message for discovery; “1” indicates the PUT request message for a binding update; “2” indicates the POST request message for registration; “3” indicates a DELETE request message, and “4” indicates the PUT request message for holding.
2049	Permanent IP address	String	128	This is the permanent IP address of the CoAP node.
2050	Temporary IP address	String	128	This is the temporary IP address of the CoAP node.
2051	Lifetime	Unit	16	This indicates the lifetime of the temporary IP address on the MMT at the MMS.
2052	Sequence number	Unit	16	This indicates the packet-sequence number, which is intended for use as a receipt identification of the last packet sequence sent by the CoAP nodes.
2053	Hold flag	Unit	1	This indicates the Hold flag, which is intended for use in hold mode.

Figure 7 shows the GET request message for discovery that retrieves the T_Addr and Lifetime of corresponding CoAP nodes. The GET request message for discovery, as shown in Figure 7, is an example of the request message of CoAP Client. CoAP Client constructs and sends a GET request message for discovery, including the P_Addr of CoAP node. As a response, CoAP Client receives the ACK response message for discovery, including the P_Addr, Cur_T_Addr, and Cur_Lifetime.

Figure 8 shows a PUT BU request and an ACK binding response. CoAP Client constructs and sends a PUT binding update request message, including P_Addr, and New_T_Addr, in option value. As a response, CoAP Client receives an ACK binding response message.

Figure 9 shows a PUT holding request and an ACK holding response message. CoAP node creates the PUT request message, including the P_Addr and H flag “1” and sends its message to CoAP Client. As a response, CoAP Client responds to the ACK response message, including the sequence number. The sequence number is intended for use as the receipt identification of the last packet sequence sent by CoAP nodes.

Figure 10 shows a message format of the POST registration request message and ACK registration response message. These messages are intended for use as the request for registering information, *i.e.*, P_Addr and Cur_T_Addr to the WMMS. In a response message, the WMMS sends the CoAP ACK message including 2.01 and message ID.

Figure 11 shows the DELETE request message and ACK registration response message. These messages are intended for use as the request to delete the IP address such as the P_Addr of the CoAP node on WMMT at WMMS.

## 4. Mathematical Analysis of Handover Delay and Packet Loss of CoMP

In this section, we mathematically analyze both handover delay and packet loss for mobility management using CoMP. In particular, we compare the performance of the proposed CoMP with that of the IETF MIPv6 and IETF HMIPv6 mobility management protocols.

### 4.1. Analysis of Handover Delay

In a WoT service environment, the handover delay and packet loss rate are important performance factors in mobility management [22]. For example, medical emergency service requires high quality in handover delay and packet loss rate.

**Figure 12:**
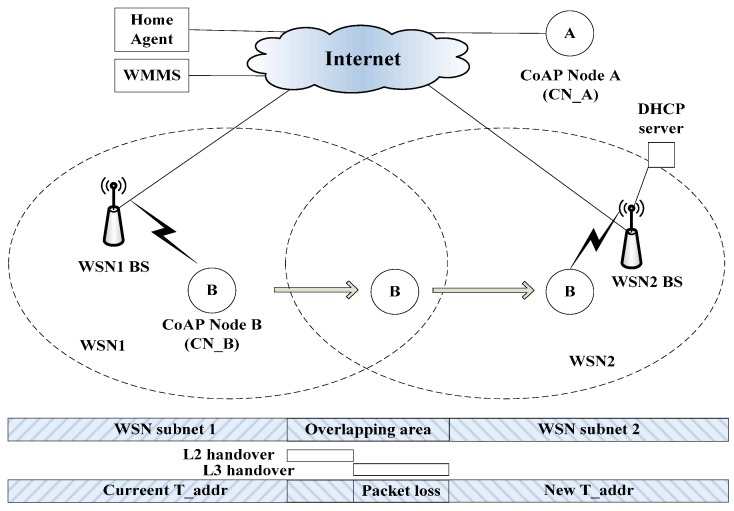
Mobility model for performance evaluation.

Figure 12 shows the mobility model for the handover delay and packet loss were analytically derived. The mobility model shows the CoAP node B in the WSN1 BS moves into the WSN2 BS. At this time, the handover latency and packet loss are measured and analyzed. The handover latency at a mobile node site is the time interval during which a mobile node cannot send or receive any packets during handover and it is composed of link layer and IP layer handover latency. In this paper, we include the handover delay timeline of the application layer during the handover. 

The mobility model consists of the CoAP node (CN_B), CoAP Client (CN_A), WMMS, HA, WSN BS1 BS, WSN BS2, and DHCP. First, CN_B connects to WSN BS1 BS and obtains the P_Addr from WSN BS1 BS. CN_B and CN_A then exchange packets. CN_B then moves into the network domain of WSN BS2, and the CN_B starts the handover procedure and obtains a new T_Addr from WSN BS2.

After this step, the mobile node performs the binding procedure and completes the handover procedure. In this handover procedure of CN_B, MIPv6, HMIPv6, and CoMP are mobility management protocols.

**Figure 13:**
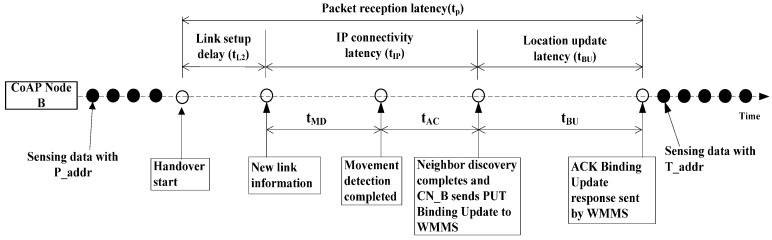
Handover delay timeline of CoMP.

The handover delay at a CoAP node side is the time interval during which the CoAP node cannot send or receive any packets during a handoff, and is composed of both L2 and L3 handover latencies [22,23,24]. Figure 13 shows the handover delay timeline caused by executing the CoMP. The white small circle indicates the time line during the handover of CoAP node between WSN BS1 and WSN BS2. The total handover delay, *i.e.*, the packet reception latency t_p_, consists of the link setup time (t_L2_), which is caused by an L2 handover; the IP connectivity latency (t_IP_); and the location update latency (t_BU_). Here, t_IP_ is the sum of t_MD_, t_AC_, and t_BU_, where t_MD_ represents the movement detection delay; t_AC_, the address configuration; DAD, the delay; and t_BU_, the BU delay between the CoAP node and WMMS. 

To analyze the delay more precisely, in the following passage, we describe the delay caused by a signaling message between the CoAP node and WMMS. Let t_X,Y_ be defined as a one‑way signaling message transfer delay between nodes *X* and *Y*. One of the endpoints is a CoAP node, and t_X,Y_ can be computed as follows:

(1) $${\text{t}}_{\text{X,Y}}(s)=(\frac{s}{B_{wl}}+L_{wl})+((d_{x,y}-1)(\frac{s+s_{t}}{B_{w}}+L_{w}+\varpi ))$$

Here, *S* is the size of the signaling message, and *B_wl_* and *B_w_* are the bandwidths of the wireless and wired links, respectively. *L_wl_* and *L_w_* are the link delays of the wireless and wired links; ϖ is the average queuing delay at each router on the Internet; *d_x,y_*−1 is the average number of hops in a wired link between nodes *X* and *Y*; and *S_t_* is the tunneling packet size. In Equation (1), the first and second terms indicate a one-way signaling message transfer delay in a wireless and wired link, respectively, between nodes *X* and *Y*. For an analytic performance evaluation, a formula for the handover latency was derived for each mobility management protocol. As described in [22], the handover latency in MIPv6 is composed of t_L2_, t_MD_, t_AC_, t_BU_, and t_RR_. Here, t_BU_ is the time delay incurred when the CN_B conducts a BU to the HA. t_RR_ is the time delay caused by executing a return routability procedure. For MIPv6, t_BU_ is equal to 2 (t_CN_B, HA_ + t_CN_B, CN_), and t_RR_ is equal to 2 (t_CN_B, CN_ + t_CN_B, HA_ + t_HA, CN_). MIPv6 uses a bi-directional tunnel between the HA and CN_B.

Because HMIPv6 is only used for local mobility management, a BU for either the HA or CN, *i.e.*, CN_A, is not necessary. However, instead of HA/CN, it requires a BU for the mobility anchor point (MAP), and thus, binding update delay, *i.e.*, t_BU_ incurs when sending signaling messages back and forth between CN_B and the MAP. It creates a handover delay of 2_CN_B, MAP_. In the case of CoMP, the handover latency is composed of t_L2_, t_MD_, t_AC_, and t_BU_. Here, t_BU_ represents a binding update signaling message delay, *i.e.*, a PUT binding update request message and an ACK binding update response message. Table 3 shows a summary of total handover delay for MIPv6, HMIPv6, and CoMP.

**Table 3:** Handover Latency.

Protocol	Total Handover Latency
*D_MIPv6_*	t_L2_+t_MD_+t_AC_+4(t_CN_B,_ _HA_+ t_CN_B,_ _CN_)+2t_HA,_ _CN_
*D_HMIPv6_*	t_L2_+t_MD_+t_AC_+2t_CN_B, MAP_
*D* *_CoMP_*	t_L2_+t_MD_+t_AC_+t_CN_B, WMMS_+t_WMMS, CN_B_+t_CN_B, CN_A_

### 4.2. Packet Loss Analysis

Packet loss is the amount of packets dropped, lost, or corrupted during transfer. Because the packet loss is proportional to the handover delay, the packet loss P*_HOprotocol_* of the handover protocol of *HOprotocol* can be calculated as follows:

P*_HOprotocol_* = λ_p_ D*_HOprotocol_* (2)

Here, λ_p_ is the packet arrival rate in packets per time units, and D*_HOprotocol_* is the handover delay of the handover protocol of *HOprotocol*. A summary of the total packet loss for MIPv6, HMIPv6, and CoMP is shown in Table 4. In the case of CoMP, a PUT holding request message and an ACK response message between the CoAP B and the WMMS are required during a handover to maintain the hold mode. Because it is assumed that during the hold mode, almost no packet loss occurs, packet loss during the handover operation is zero.

**Table 4:** Packet Loss Analysis.

Protocol	Total Packet Loss
P*_MIPv6_*	λp *D_MIPv6_*
P*_HMIPv6_*	λp *D_HMIPv6_*
P*_CoMP_*	Zero

## 5. Performance Evaluation

### 5.1. Simulation Configuration

In this subsection, we present the simulation configuration environment to simulate the proposed CoMP handover mechanism. We used an OMNeT++ network simulator [25], which runs on a Linux operating system. We compared the handover performances of MIPv6, HMIPv6, and CoMP.

**Figure 14:**
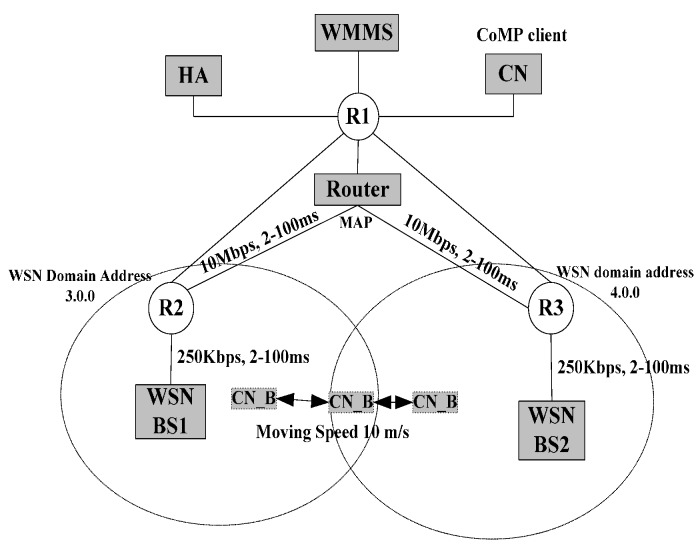
Simulation topology and parameter.

Figure 14 shows the network topology used in our simulations for MIPv6, HMIPv6, and CoMP. This topology has been used extensively for mobility management performance studies. The coverage of WSN BS was set to 50 m within a 200 m × 200 m area. It is assumed that once a CoMP sensor node moves out of the coverage of WSN BS1 BS, the new T_Addr is available. The following configurations were used in simulation:

• To stabilize the results, each simulation of the linear back and forth movement between WSN BS1 BS and WSN BS2 of the CoAP node lasted for 100 s.
• The IEEE 802.15.4-2006 standard is used for the MAC layer, and each WSN BS has a radio coverage area radius of approximately 20 m. The overlapping region between WSN BS1 BS and WSN BS2 is 5 m. The advertisement period of the HA/WSN BSs is 1 s, although the advertisements are not synchronized.

Table 5 shows the basic system parameters for evaluating the performance of the mobility management protocols. Most parameters in this analysis were set to typical values found in [21,22,23]. We use the parameters in Table 5 to analyze the mathematical performance evaluation of the handover latency and packet loss during the handover procedure described in Section 4.2.

**Table 5:** Simulation Parameters.

Parameter	Symbols	Value
Auto-configuration delay	t_AC_	500 ms
Movement detection delay	t_MD_	100 ms
L2 setup delay	t_L2_	50 ms
Wired-link bandwidth	B_w_	10 Mbps
Wireless-link bandwidth	B_wl_	20~250 kb/s
Average queuing delay	ϖ	0.1 ms
Wireless-link delay	L_wl_	15 ms
Wired-link delay	L_w_	2 ms
Control packet size	*S*	50 bytes
Tunnel packet	*S_t_*	80 bytes
Packet arrival rate	*λ_p_*	Default value 10 packets/s
Average speed of node	*V*	10 m/s

In Table 5, the *auto-configuration delay* indicates the time interval during the duplicate address detection procedure, and the *movement detection delay* indicates the time interval during which the CoAP node recognizes whether the current network domain is in the same domain. The L2 handover delay indicates the time interval during the link layer handover procedure. Section 4.1 provides further description of these parameters. It is assumed that the number of hops between the CoAP node and WSN BS, between the CN and HA, between the WMMS and WSN BS1 BS/WSN BS2, and between the HA/CN and WMMS are set to 1, 2, 2, and 2, respectively. In the performance evaluation, we used UDP-based Constant Bit Rate (CBR) traffic with bit rates of below 56 Kb/s, a packet size of 1024 bytes, and a packet arrival rate under 55 packets/s.

### 5.2. Performance Results and Analysis

**Figure 15:**
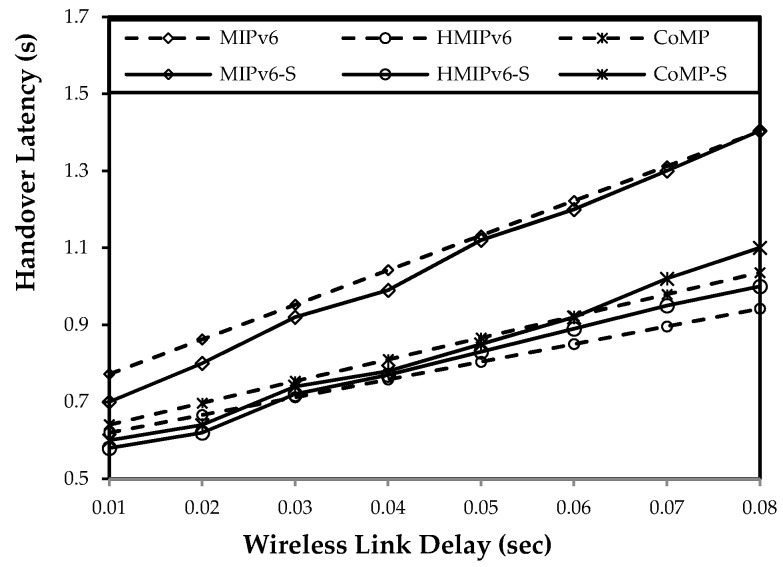
Impact of wireless link delay on handover latency.

**Figure 16:**
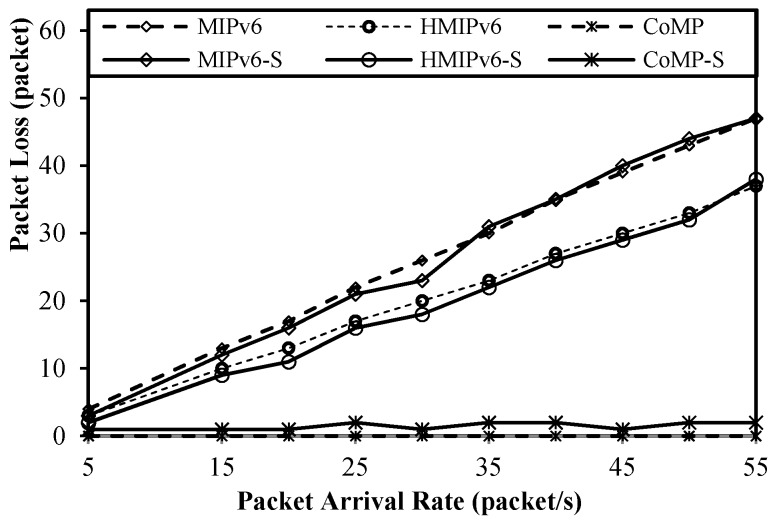
Packet loss as a function of packet arrival rate.

**Figure 17:**
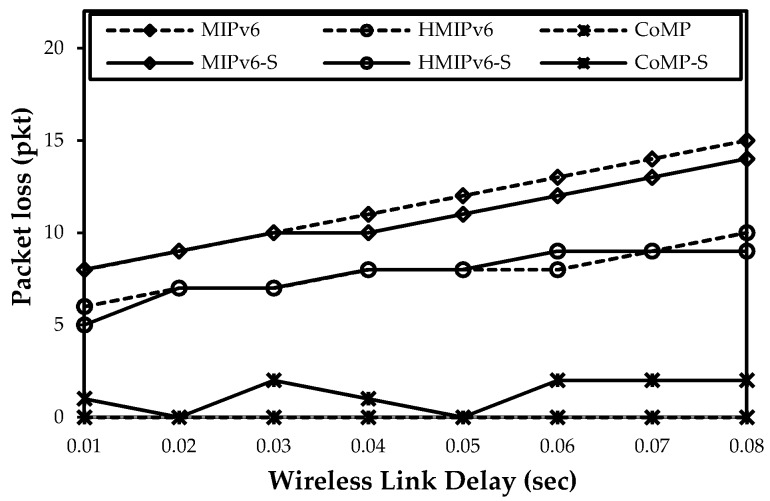
Impact of wireless link delay on packet loss.

Figure 15, Figure 16 and Figure 17 show both mathematical analysis and simulation results. In these figures, a continuous line indicates the simulation results, and a dotted line indicates the mathematical analysis results. We analyze the handover latency, packet loss, signaling cost, and power consumption.

### 5.3. Handover Latency Analysis

Figure 15 shows the change in handover latency of the mobility protocol based on changes in the wireless link delay. The handover delay can be as large as the number of the control packets during the handover between the WSN BS and CoAP node increases. As Figure 15 shows, the handover latency of the proposed CoMP is similar to the results of the HMIPv6. For HMIPv6, a BU message is exchanged between the CoAP node and MAP. In contrast, for CoMP, a PUT BU request message and an ACK BU response message are exchanged between the CoAP node and WMMS.

### 5.4. Packet Loss Analysis

Figure 16 shows the change in packet loss in terms of the packet arrival rate. The packet loss rate is important in service reliability in the WoT monitoring service [12]. As Figure 16 shows, the packet loss of the proposed CoMP is less than that of MIPv6 and HMIPv6. The packet loss of both MIPv6 and HMIPv6 increases sharply as the packet arrival rate increases. In contrast, almost no packet loss occurs for CoMP because the protocol uses the hold mode of operation.

Figure 17 shows the impact of packet loss with regard to a variety of wireless link delays. To measure the packet loss with regard to the wireless link delay, the value of λ_p_ was set to 10 packets/s; *L_WL_* was set to 0.002 s; and *L_W_* was set to vary between 10 ms and 80 ms. In MIPv6 and HMIPv6, packet loss increases as *L_W_* increases. However, in CoMP, the amount of packet loss is less than that of HMIPv6 and MIPv6 under the conditions of varying wireless link delays. In CoMP, the PUT holding mechanism can be dynamically reduced. The results of both the mathematical analysis and the simulation are almost the same.

## 6. Conclusions

This paper has discussed a reliable and seamless mobility support scheme for IoT sensor nodes, which are extremely energy and resource constrained devices in nature, *i.e.*, less CPU processing, low memory, and power supply capabilities than Internet devices. A variety of IoT services have been attempted, e.g., healthcare monitoring services, public transport vehicle service, V2I automatic vehicle networks, home networks, automotive networks, automatic systems, industrial networks, interactive toys, and remote meters. Specifically in the healthcare service, the reliable data transmission of vital sensing data is very important while the mobile sensor node moves into different wireless network domain. To guarantee the reliable data transmission, the reliable mobility management protocol is required while considering the characteristics of the constrained device. We proposed a mobility management protocol named CoMP, which can make the sensing data of sensor nodes to be retrieved effectively, while IoT sensor nodes are moving. The salient feature of CoMP is that it makes use of the IETF CoAP protocol at application layer for mobility management, instead of using Mobile IP at network layer. It can eliminate additional signaling overhead of Mobile IP, providing the reliable mobility management, and preventing the packet loss. We have designed the architecture, message formats and detailed signaling procedures of CoMP. More specifically, the IETF CoAP message formats are extended for supporting the registration, discovery, binding and holding operation. Finally, by both mathematical analysis and simulation, we have conducted comparative performance evaluation between CoMP and MIPv6/HMIPv6 in terms of handover latency and packet loss. The results show that the proposed CoMP is superior to previous mobility management protocols. Further work may be required to be done on the security issues related to CoMP.
